# Review of Recent Computational Research on the Adsorption of PFASs with a Variety of Substrates

**DOI:** 10.3390/ijms25063445

**Published:** 2024-03-19

**Authors:** Alfonso Minervino, Kevin D. Belfield

**Affiliations:** Department of Chemistry and Environmental Science, College of Science & Liberal Arts, New Jersey Institute of Technology, 323 MLK Blvd., Newark, NJ 07102, USA; am2923@njit.edu

**Keywords:** PFASs, adsorption mechanisms, computational chemistry, density functional theory (DFT), molecular dynamics (MD)

## Abstract

The widespread use and impervious nature of per- and polyfluorinated alkyl substances (PFASs) is leading to potentially harmful exposure in numerous environments. One avenue to explore remediation of PFAS-contaminated environments involves investigating how well PFASs adsorb onto various substrates. In the current review, we focus on summarizing recent computational research, largely involving density functional theory (DFT) and molecular dynamics (MD), into the adsorption and interaction of PFASs with a variety of substrates with an aim to provide insight and inspire further research that may lead to solutions to this critical problem that impacts the environment and human health.

## 1. Introduction

Per- and polyfluorinated alkyl substances (PFASs) are a class of synthetically made compounds with multiple fluorine atoms attached to the main carbon chain. These fluorine atoms replace hydrogen atoms attached to carbon, creating a more electronegative environment around the molecule. The environment yields more hydrophobic and lipophobic properties for the molecule, creating compounds that are extremely resistant to any type of chemical degradation, enabling these molecules to be utilized in a vast number of products spanning multiple industries since their discovery in the 1940s [1,2]. Some common uses of PFASs that have been incorporated over the years include nonstick coatings on cookware, water-repellant cloth, and aqueous film-forming foams (AFFFs) used in firefighting, packaging, and cosmetics [1,2,3]. In all these applications, PFASs perform extraordinarily well, and thus the use of these materials has expanded [4]. However, as these products were used and disposed of, PFASs inevitably entered the environment where the properties that made these compounds so resilient and desirable in products caused these compounds to become persistent in the environment. PFASs do not naturally break down in the environment because of the strong C-F bonds that are throughout the structure and have become known as “forever chemicals” [5,6].

As these PFASs continue to collect and persist in the environment, more and more ecosystems are being exposed to increasing levels of PFAS contamination. Studies have shown that PFAS contamination can be found in drinking water, surface and groundwater, air, sediments, soil, and several aquatic species [6,7,8,9]. In turn, this contamination has worked its way into food supplies and has been shown to, inevitably, bioaccumulate in plants, animals, and humans [10,11,12]. Regarding humans, PFAS bioaccumulation has been linked with several adverse health effects including carcinogenic effects, neurotoxicity, cardiovascular disease, developmental issues, and reproductive issues [13,14]. Although current research is still investigating all the potential adverse effects of PFAS bioaccumulation, PFAS contamination and removal have been widely recognized as issues that need to be addressed. As a result, PFAS regulations have begun to be established in numerous countries worldwide [15].

One way of addressing this ongoing issue is using adsorbents to physically remove PFASs from various water supplies. Experimental evidence using various adsorbents has shown promise to varying degrees, but challenges arise due to the relatively low concentrations of PFASs in some sources as well as the propensity of PFASs to resist chemical reactions [16,17,18,19,20,21,22,23]. Computational methods can be utilized to complement ongoing experimental findings or even to drive new research to find novel materials, and have been increasing in complexity given the enormous growth of computing power over the last few decades. Systems can be established that include PFASs of all types in different conformations interacting with a wide range of materials with a variety of characteristics to determine if favorable PFAS adsorption occurs. The benefits of using computational methods include the ability to investigate a wide range of systems and materials in a relatively short amount of time and working with simulations that include optimized structures and conditions. Provided that the simulations are not too computationally expensive, the results can be acquired and analyzed for materials where the material cost is expensive or the experimental procedure to synthesize the material is too complicated or has not yet been optimized. However, this could be seen as a drawback for computational studies as well. These systems are optimized under certain conditions and may not precisely reflect real-world conditions at contaminated sites. Although it may not be definitive in terms of providing concrete evidence for a particular question, when paired with experimental results, computational studies can provide critical insights into questions regarding PFAS adsorption [24,25].

Two of the most common computational methods that can accurately and efficiently determine adsorption energies in various systems include density functional theory (DFT) and molecular dynamics (MD). DFT uses the electron density of the given system as the defining quantity of the system and can determine atomic forces and energies of the system by approximating the Schrödinger wave equation. The results of these calculations give insight into how materials behave when interacting with each other. The advantages of DFT-based calculations include more accurate and reliable results while examining the electronic structure of the system, for example, the electron density of a single PFAS molecule interacting with one layer of the substrate surface. These calculations, however, can be computationally more expensive than classical MD calculations and can be limited in the size of systems that can be efficiently analyzed; moreover, they do not take dispersion forces into account if corrections or other optimized functionals are not used [26]. Dispersion interactions can be accounted for using nonlocal density functionals (such as vdW-DF1), semiclassical *C_6_*-methods (such as D3), or one-electron effective potentials (such as atom-centered external potentials) [27,28,29,30,31]. MD analyzes the dynamics of the nuclei of the atoms in a system using classical equations of motion, without taking the electrons into effect. This method can analyze simulations of large-scale systems for up to several nanoseconds with thousands of atoms and can shed light on the mechanism driving a particular interaction. Classical MD simulations are less computationally expensive, which can allow for larger systems to be studied across longer timescales, such as a solvated system of PFASs at naturally occurring concentrations interacting in the porous structure of a substrate. As systems get larger, some loss of the finer details of the system can occur. Approximations of the effects of electrons, taken as potential energy surfaces, are also required [26,32,33].

Enhancements to these methods, such as coarse-grain MD, dissipative particle dynamics (DPD), and ab initio MD (AIMD), aim to make calculations easier, more efficient, or more accurate. Coarse-grain MD utilizes the condition that some groups of atoms are treated as a single atom. Generalized parameters for the groups of atoms can allow for simulations to be carried out for much longer, up to the microsecond timescale, and can be a good approximation for extremely large systems, such as proteins and carbon nanotubes [34,35,36], although there have been no studies directly examining PFAS adsorption using coarse-grain MD. Other studies that use coarse-grain MD when investigating PFASs examine PFAS aggregation in solvents used to manufacture membranes, fuel cells, and catalyst inks [37,38,39]. DPD incorporates short-range, soft repulsion potentials while simulating the dynamics of the system, which lends itself to higher time steps than MD [40]. AIMD may utilize DFT methods or other correlated wavefunction methods such as Møller–Plesset theory or coupled cluster methods to provide a more realistic analysis of the system to more closely resemble experimental conditions [41,42,43,44,45]. This can provide even more precise results but can vastly increase the computational cost and significantly limit the practical timescale, up to only a few picoseconds, for a given level of detail [33,46].

Each of these methods is very powerful in determining critical information regarding a system and they can be used in a complementary fashion with each other as well as with experimental results to come to a better understanding of a particular question. The acquired results can be comparable between the methods, provided that the level of detail and appropriate approximations are used [47,48,49]. Although there has been some research into the degradation mechanisms and pathways of PFASs [5,50], in this review paper, we focus on summarizing recent computational research into the adsorption of PFASs using a variety of substrates. Most of the research, both experimentally and computationally, has focused on two specific PFAS compounds, perfluorooctanesulfonic acid (PFOS) and perfluorooctanoic acid (PFOA), as well as their conjugate bases due to these being the most manufactured (now phased out of production) and environmentally detected PFASs [51,52]. Other PFAS compounds have also been studied to gain a better sense of the levels of adsorption across different types of PFASs featuring different characteristics, including perfluorobutanesulfonic acid (PFBS) and perfluorohexanesulfonic acid (PFHxS), though many more have been identified [53]. Broadening the amount of PFAS research will be critical to understanding the different levels of adsorption necessary to address the growing PFAS contamination issues affecting global ecosystems.

## 2. Adsorbents and Morphology

### 2.1. Activated Carbon

Some of the most common PFAS adsorbents currently in use include activated carbon (AC) in the form of either granular activated carbon or powdered activated carbon. These materials can have very high surface areas and can be used to remove many different types of pollutants from water sources. Regarding adsorbing and removal of PFASs using mainly carbon-based materials, Saha et al. [54] indicate that better adsorption of PFASs would be driven by large porosities and by designing processes that more accurately reflect real-world conditions. In addition, enhancing the material to be more selective toward adsorbing PFASs through increasing the fluorophilicity and hydrophobicity properties and introducing positive charges may yield better results. Increasing porosity would improve the overall kinetics of PFAS adsorption by increasing the diffusion of large PFAS molecules into the material. Metal ions can be added as positive charges, while fluorinated moieties in the material structure would increase the overall fluorophilicity [54]. Park et al. [55] argue that calculated total negative charges showed a better correlation than hydrophobicity to the equilibrium constants. For example, the DFT calculations showed that sulfonic PFASs induced a greater negative atomic charge per oxygen atom compared with carboxylic PFASs due to the available resonance stabilization. This caused nearly double the amount of negative charge from the total oxygen atoms, which led to greater adsorption compared to carboxylic PFASs. Longer chain lengths can lead to greater adsorption capacities as well, due to the increased number of fluorinated groups. DFT calculations and MD simulations were used to examine and analyze all these characteristics and how they interplay with each other [55].

Household point-of-use water purifiers comprising coconut shell AC can achieve anywhere between 21–99% PFAS removal across 14 different PFAS materials found in tap water, as per He et al. [56]. Characterizations and several chemical analyses showed that physical adsorption, mainly through van der Waals forces and hydrophobic interactions, can reduce the amount of up to 23 different PFAS compounds from contaminated water sources. DFT calculations were conducted to help investigate the mechanism where PFASs and AC were used to estimate the adsorption energies between PFAS molecules of different lengths and head groups and the simplified AC structure of a double layer of graphite. These optimized calculations resulted in structures where the best configuration aligns the carbon chain parallel to the AC surface with the head group at an optimized distance away. The theoretical adsorption energies ranged from −0.574 eV to −1.789 eV, indicating relatively weak but spontaneous adsorption driven by enthalpy. The more negative, and therefore more favorable, adsorption energies corresponded to longer chain PFAS molecules, which falls in line with experimental results. Before adsorption, the calculations show that the PFASs are slightly negatively charged while the AC is neutral. However, after adsorption the PFASs become positively charged while the AC is negatively charged, resulting in a neutral charge overall. This reduces the band gap between the materials and makes them more conductive.

**Figure 1:**
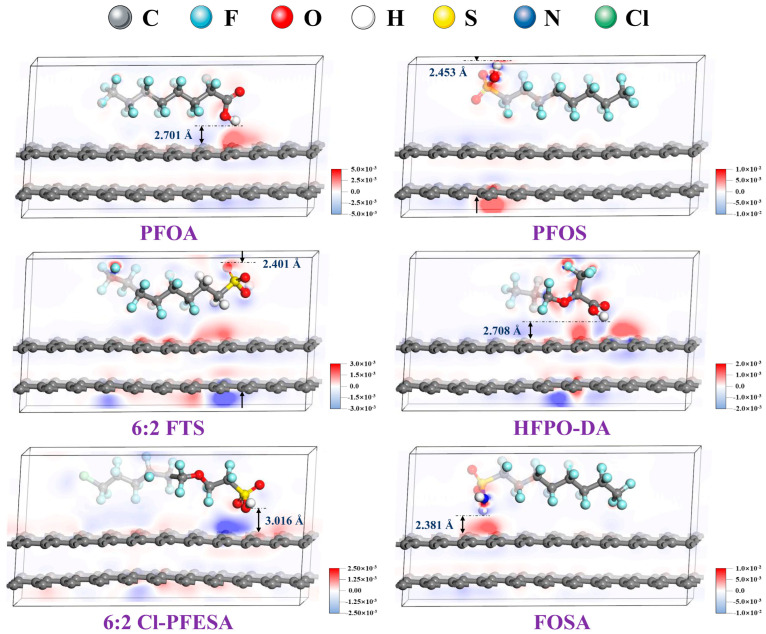
Density functional theory (DFT) calculation results showing electron density configurations of various per- and polyfluorinated alkyl substances (PFASs) with activated carbon (AC) surfaces. Electrostatic interactions between PFASs and AC are shown where red and blue indicate electron-gain and electron-loss zones, respectively. The distances shown and indicated with arrows are the shortest of the adsorption layer. Reprinted from He et al. [56], with permission from Elsevier.

Electron density configurations based on Hirshfeld atomic population analysis, as illustrated in Figure 1, show that charge transfer occurs between the head group of PFASs and the AC surfaces. The density of state (DOS) was also calculated for the components and when it was combined with all the previous calculations, the authors proposed that the energy of the system decreased after PFAS adsorption and the shortest distance between the PFAS and the AC surface is greater than the covalent bond length. This indicates that the interaction between PFASs and AC is a physical interaction with relatively weak adsorption energies where electrostatic adsorption plays an important role [56].

Yuan et al. [57] used MD simulations to investigate the role of nanobubbles in removing PFASs from aqueous environments using AC. These simulations began with investigating how a single PFOS molecule in bulk water interacts with the air–water interface in a 100 ns MD simulation. The PFOS moved toward the interface quickly with the sulfonate group acting as an anchor preventing the PFOS from leaving the water through hydrogen bonds between the sulfonate group and water molecules. Three additional common PFAS molecules (PFOA, PFHxS, and PFBS) were also initially investigated using the same 100 ns MD simulation. The carbon chains for each moved outside the bulk water and into the air layer of the interface, while the movement through the bulk water was monitored throughout the simulation by plotting the z-coordinate of the position over the course of the simulation. Plotting the position for each PFAS showed that all PFASs moved to the air–water interface region within the first 8 ns of the simulation indicating that PFASs would be concentrated in this area when nanobubbles or foam entered the mixture. Next, the interface properties of PFASs were examined using a characteristic angle, defined by Yuan et al. as the angle between the vector of the S1/C8 atom to the C1 atom and the interface normal. Using this measurement across the four different PFASs investigated, PFOS had the narrowest range of angles calculated, indicating that PFOS was the most ordered at the interface region. PFOS also had the largest characteristic angle among the four tested followed by PFOA, then PFHxS, then PFBS. This was attributed to the hydrophobicity of the chains increasing as the chain length increases, resulting in higher characteristic angles [57].

**Figure 2:**
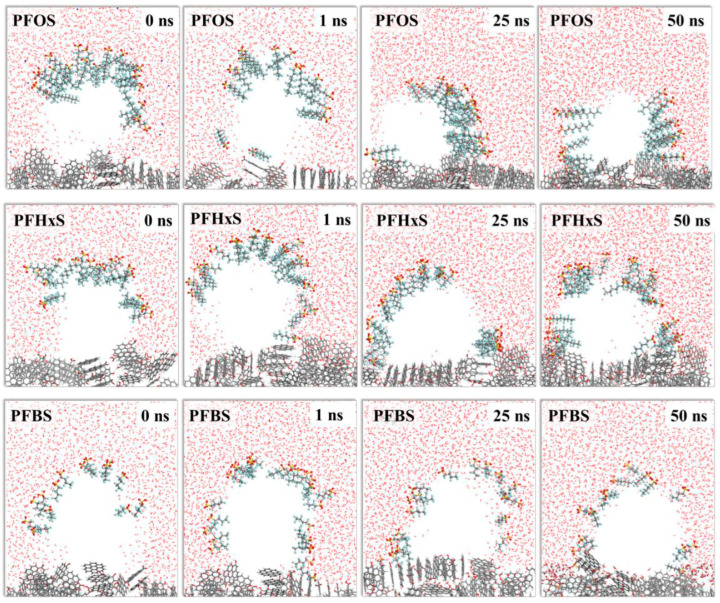
Molecular dynamics (MD) simulations of PFASs at certain time intervals illustrating nanobubbles that form to help adsorb PFASs onto the AC surface. Black = C, red = O, white = H, yellow = S, and light blue = F. Reprinted from Yuan et al. [57], with permission from Elsevier.

Other studies have also found that higher characteristic angles lead to greater stability of nanobubbles that form, as noted by Zhang et al. [58]. Gibbs free energy calculations, conducted by Yuan et al. [57], supported the notion that PFOS was able to reach the interface surface more effectively than the other three PFASs tested, ranging from −3.32 kcal/mol for PFBS to −6.70 kcal/mol for PFOS. MD simulations were then used to investigate the stability of the formed nanobubbles of the PFASs and these simulations found that the PFOS nanobubbles had the highest viscosity and thickness, indicating that the PFOS liquid film was the most stable of the four PFASs. Nanobubbles can easily exist on the surface of AC and are able to capture PFASs that accumulate on the surface so further MD simulations were carried out to investigate the air–water–AC three-phase interface. Based on the free energy barriers previously calculated, there were multiple mechanisms of movement for the tested PFASs and how they were captured by the AC and nanobubbles. Smaller free energy barriers for PFBS, for example, caused PFBS to move along the interface region as well as leave the interface region and migrate into the water phase, making it more difficult for AC to capture these PFASs. For higher free energy barriers, like for PFOS, this caused the PFOS to only move along the interface surface before being captured, making it easier for the AC to capture these PFASs. Figure 2 shows the MD simulations at certain time intervals and how the PFASs are captured by AC and form the three-phase interface. To begin the simulations, the nanobubble is on the surface of the AC with several PFAS molecules in the bulk water phase. As time progresses, the PFAS molecules are captured by the nanobubbles to varying degrees, corresponding to the free energy barriers, and are adsorbed onto the AC surface, as described above. Interaction energies and electrostatic potentials were also calculated to understand the mechanism of PFAS adsorption onto AC. These results show that the PFAS anions were able to effectively interact with the positively charged AC and form electrostatic interactions. Modifying the AC to have a greater overall positive charge could enhance the effectiveness of AC to adsorb PFASs with the use of foam-generating agents that can reduce the surface tension of water and introduce more opportunities for nanobubbles to form [57].

### 2.2. Graphene

As reported by Jiang et al. [59], DFT and MD were used to explore how nanobubbles impact the adsorption of PFASs onto slightly different carbon-based materials, pristine and functionalized graphene (GR). The following three materials were prepared and investigated both experimentally and computationally: GR, graphene functionalized with NH_2_ (GR-NH_2_), and graphene functionalized with OH (GR-OH). The amino groups were used to mainly represent electrostatic interactions while the hydroxyl groups represented hydrogen bonding interactions. Initial DFT calculations looking at adsorption energies showed that GR-NH_2_ had a much higher affinity for various PFASs than GR-OH, while GR had the lowest affinity calculated. The electrostatic potentials calculated for each of the structures showed that the amino groups were protonated and electrophilic in neutral conditions while interacting with the negatively charged sulfonate head groups. On the contrary, the hydroxyl groups were not charged and formed mostly hydrogen bonds with the charged head groups. For GR, there was virtually no charge on the structure so the PFASs could not adsorb directly. MD simulations progressed from systems without water to systems with water and, finally, to systems with nanobubbles in water. In simulations without water, the head groups of the PFASs oriented themselves near the functionalization on GR but were in an irregular fashion with nearly no interactions on pristine GR. Radial distribution function (RDF) calculations from these simulations confirmed the results. In the MD simulations with water, the same results for GR-NH_2_ were found but were slightly different for GR-OH. In this case, with PFOS specifically, it entered the liquid phase near the end of the simulation as hydrogen bonds became the main interactions, as opposed to electrostatic interactions. For simulations that included the nanobubbles in water, the results were similar for GR regardless of whether the nanobubbles were located on the surface of the GR or dispersed in solution. The simulations for GR showed that the nanobubbles ended up adhering to the surface of the GR with PFOS head groups remaining in the water phase with CF chains extending into the air phase of the nanobubble. The nanobubbles can enrich the PFOS and be adsorbed onto the GR more effectively.

**Figure 3:**
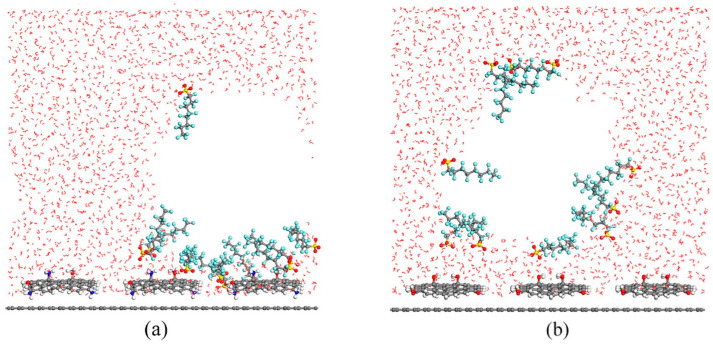
Snapshots at the end of perfluorooctanesulfonic acid (PFOS) MD simulations with water and nanobubbles interacting with functionalized graphene (GR). (**a**) Graphene functionalized with NH_2_ (GR-NH_2_) and (**b**) graphene functionalized with OH (GR-OH). Reprinted (adapted) with permission from Jiang et al. [59]. Copyright 2021 American Chemical Society.

Regarding the nanobubbles and their interactions with the functionalized GR, slightly different mechanisms drove the interactions. Figure 3 shows a snapshot at the end of the MD simulations for each of these functionalized systems. For GR-NH_2_, the nanobubbles could not adhere directly onto the surface, but the PFOS, through the strong electrostatic interactions, was shown to drag enriched nanobubbles to the surface of the GR-NH_2_ and enhance PFOS adsorption overall. For GR-OH, the nanobubbles also could not adhere directly to the surface. However, the adsorption was not enhanced as hydrogen bonds were the main interactions in this system [59].

### 2.3. Smectite Clay

**Figure 4:**
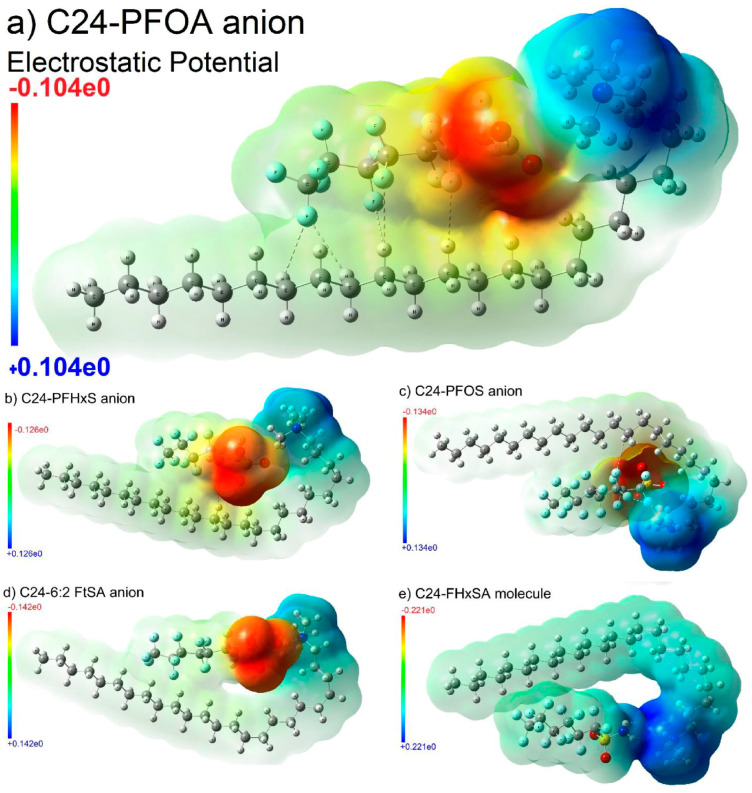
DFT-optimized electrostatic potential maps of various PFASs interacting with quaternary ammonium surfactant. Reprinted from Yan et al. [48], with permission from Elsevier.

Several other materials can be used for the adsorption of PFASs, including naturally occurring materials such as smectite clay. Modifying this material by inserting cations such as quaternary ammonium surfactants in the exchange sites of the clay results in surfactant-modified clay (SMC). This material has been shown to meet or exceed the performance of AC for the removal of PFASs from contaminated water sources. As reported by Yan et al. [48], adsorption experiments and scanning transmission X-ray microscopy (STXM) paired with X-ray absorption near-edge structure spectroscopy (XANES) showed that the adsorption of PFASs by SMC varied with small changes in concentration and the mechanism involving the adsorption sites could change based on the concentration as well. The STXM-XANES spectroscopy results also suggested that PFOA enters the interlayer space of SMC and forms electrostatic interactions with quaternary ammonium species. All-atom MD simulations and DFT calculations indicated that PFASs interacted with the positively charged surfactants through charge–charge, charge–dipole, and dipole–dipole interactions. Of the PFAS compounds tested, PFOS showed the strongest interactions, indicating the SMC may have the highest adsorption capacity for PFOS. Figure 4 shows the DFT-optimized electrostatic potential maps of some of the tested PFASs with a quaternary ammonium surfactant. Electrostatic interactions as well as the dipole interactions (dashed line) with the labeled atoms can be seen. The adsorption energies were calculated using a thermodynamic cycle utilizing DFT calculations. The absolute values of the energies calculated increased with increasing chain length due to the hydrophobicity increasing as chain length increases. Depending on the PFAS structure, the mechanism may vary but the interactions with the positively charged surfactants overcome the interaction with water and allow access to the interlayer space [48].

**Figure 5:**
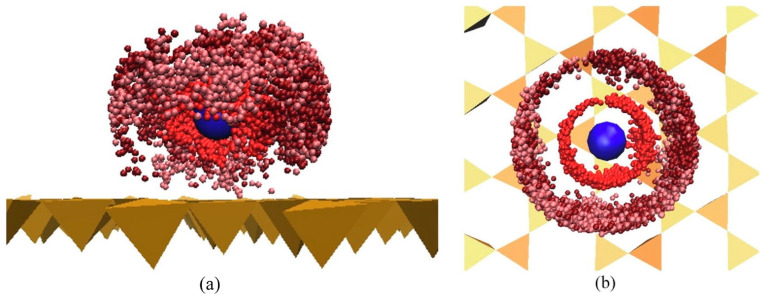
Edge-on view (**a**) and top-down view (**b**) of perfluorobutanesulfonic acid (PFBS) (red and purple) adsorbed onto smectite clay surface (yellow) in the presence of Ca^2+^ (blue). Reprinted from Willemsen and Bourg [60], with permission from Elsevier.

In another study on smectite clay, Willemsen and Bourg [60] simulated a system of a stack of flexible smectite layers in contact with bulk water containing PFASs. This system was analyzed using MD analysis to investigate the effects of PFAS size and coordinating cation type on adsorption. Of the cations that were tested, K^+^, Na^+^, and Ca^2+^, the partition coefficient was most enhanced in the presence of Ca^2+^. This was consistent with previous experimental results, indicating that a cation bridging phenomenon occurs as a divalent cation can bind to two unique anionic PFASs to increase the potential adsorption. Atomic density profiles indicated that the CF chains displace water from the first hydration layer of the clay and lie flat on the surface in hydrophobic areas, minimizing the interactions between the hydrophobic tails and water. The hydrophilic head groups are oriented away from the clay surface to maximize interaction with water. As shown in the MD simulations, the head groups also tended to coordinate with Ca^2+^ to form bridging interactions with the anionic species. Figure 5 shows how PFBS adsorbs onto the smectite surface. The CF tails, in this domain, displaced the first layer of water around the cation and formed an inner sphere complex with the cation and surface. Overall, the enthalpy of adsorption is unfavorable, but much less so in the presence of calcium cations, as they minimize Coulombic repulsion. Regarding the PFAS size related to adsorption onto smectite clay, the simulations suggested that the entropic adsorption of long-chain PFASs increases linearly with chain length. However, for short-chain PFASs, smaller sizes of the structure can lead to more PFASs accessing hydrophobic sites and coordinating with calcium cations [60].

### 2.4. Rutile TiO_2_

In multiple studies from He et al. [61,62], MD simulations were carried out using PFOS and its interactions with rutile. Rutile is the most abundant form of TiO_2_ and is one of the most common metal oxides in industry today. The simulation carried out showed that the PFOS formed a compact layer on the surface with structures that varied based on the crystal structure of the surface. On the (110) and (001) rutile surfaces, PFOS mainly attached through the sulfonate head group. However, differences were apparent as a monolayer formed on the (110) surface with the CF chains nearly perpendicular to the surface. The (001) surface exhibited the CF chains to be at a 30–75° angle to the surface and formed a hemicylinder configuration.

**Figure 6:**
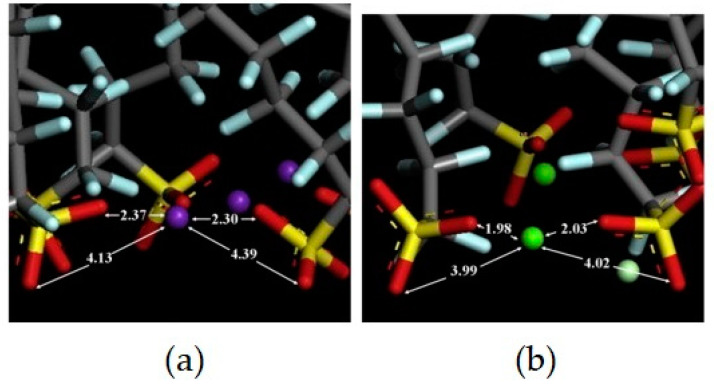
PFOS sulfonate head groups interacting with bridging ions: (**a**) K^+^, in purple, and (**b**) Ca^2+^, in green. All distances are noted in Angstroms. Reprinted from He et al. [61], with permission from Elsevier.

In addition, the PFOS formed an irregular pattern on the (100) surface as both the sulfonate head group and the CF chains interacted with the surface, the former via electrostatic interactions and the latter via van der Waals interactions. As the concentrations of PFOS varied, the simulations found that the PFOS mainly directly interacted with the rutile surface through electrostatic interactions at low and intermediate concentrations. At high concentrations of PFOS, the PFOS aggregated into a multi-layered structure on top of the already formed monolayer through van der Waals interactions between the CF chains. When introducing CaCl_2_ into the system, the multi-layered structure converted back into a monolayer structure. The divalent Ca^2+^ replaced the monovalent K^+^ as the bridging ion and formed stronger interactions with the sulfonate groups of the PFOS, evidenced through closer interaction distances, as shown in Figure 6. This indicated that the addition of Ca^2+^ increased the critical concentration of PFOS to begin to form multi-layered structures [61,62].

### 2.5. Chitosan

**Figure 7:**
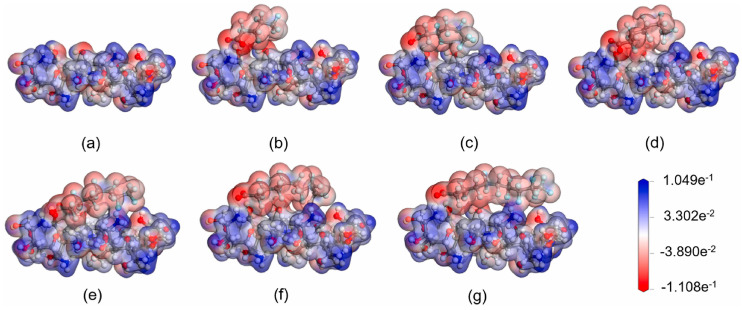
Electrostatic potential maps of (**a**) chitosan, (**b**) chitosan with perfluoropentanoic acid (PFPeA), (**c**) chitosan with perfluorohexanoic acid (PFHxA), (**d**) chitosan with perfluoroheptanoic acid (PFHpA), (**e**) chitosan with perfluorooctanoic acid (PFOA), (**f**) chitosan with perfluorononanoic acid (PFNA), and (**g**) chitosan with PFDA. Reprinted from Liu et al. [63], with permission from Elsevier.

Chitosan is another naturally occurring material that has shown effectiveness in removing PFASs from aqueous environments. Liu et al. [63] employed DFT methods to calculate adsorption energies between various PFASs and chitosan. The adsorption configurations were also optimized and calculated using electrostatic potential maps. These electrostatic potential maps showing chitosan interacting with many PFASs are illustrated in Figure 7. Of all the PFASs tested, all adsorption energies were found to be positive, indicating an exothermic process with stable structures. As the CF chain length increased through the series of PFASs tested, the adsorption energy also increased, suggesting that the hydrophobic CF chains were interacting with the hydrophobic chitosan surface, in addition to the electrostatic interactions with the head groups of the PFASs and amino groups on the chitosan surface.

At low pH, adsorption increased dramatically experimentally, showing that more electrostatic interactions with the protonated amino groups were occurring. These interactions were also apparent in the DFT calculations as many protonated groups were noted in the electrostatic potential maps. The PFASs may also contribute to protonation, further increasing the adsorption of PFASs onto the protonated surface. The amino groups are more likely to be protonated than the hydroxyl groups on the chitosan surface resulting in these sites having a higher affinity for PFASs and being far more likely to participate in the electrostatic interactions, which is the main mechanism driving the adsorption in this system. Also, even though adsorption energies increased as the length of the CF chains increased, these energies reached a limit. As the CF chains move closer to the chitosan surface, they interfere with other potential electrostatic binding sites and impact the overall adsorption process [63].

### 2.6. Micelles

PFASs tend to form micelles in aqueous environments that could block the pores of different adsorbent materials. As shown experimentally by Zhang et al. [64], crosslinked chitosan beads showed high sorption capacity for removal of PFOS from aqueous solution, especially at low pH. At pH 3, nearly all the amine groups of the chitosan were protonated and interacted with PFOS through electrostatic interactions. Some hydrophobic interactions also occurred. As more PFOS entered the pores of the chitosan beads, the concentration of the PFOS increased and exceeded the critical micelle concentration (CMC) of PFOS. As more and more sites were taken on the chitosan surface, micelles, hemi-micelles, and bilayers of PFOS formed on the surface, further increasing the amount of PFOS adsorbed [64].

**Figure 8:**
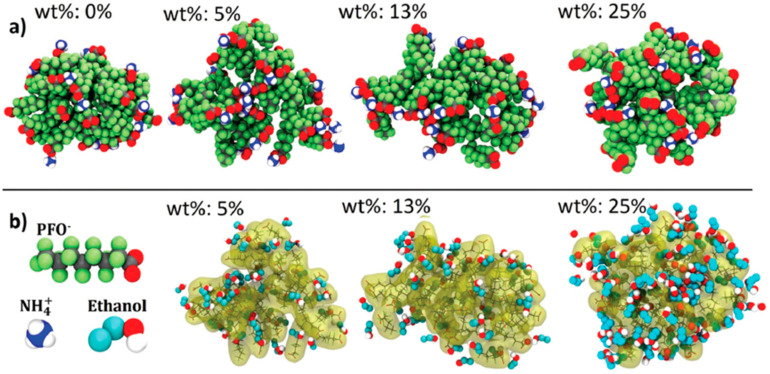
Morphologies of PFOA micelle at various concentrations of ethanol and the distribution of (**a**) NH_4_^+^ counter ions and (**b**) ethanol. Used with permission of the Royal Society of Chemistry, from Dong et al. [65]; permission conveyed through Copyright Clearance Center, Inc.

Dong et al. [65] aimed to complete experimental and computational studies of PFOA micelles with varying levels of ethanol present. MD simulations were used to investigate how these micelles changed in different environments. The system contained enough PFOA molecules to correspond to a PFOA concentration of 390 mM, which is higher than reported CMC values for PFOA at 298 K. NH_4_^+^ was used as the counter ion to balance the negatively charged PFOA ion. Other MD simulations with a scaled dipole moment on the OH bond of the ethanol represented the effects of different additives in the environment. In pure water, the simulations showed that the PFOA micelles were slightly ellipsoid in shape but fairly compact in size. However, in increasing ethanol concentrations, the micelles tended to become more diffuse and break into smaller clusters. Figure 8 shows the PFOA micelle and its morphologies as the ethanol concentration increased. This showed that monitoring and tailoring the formed micelles in solution to specific sizes and shapes could help prevent them from blocking pores and reducing the overall adsorption effectiveness of different materials [65].

**Figure 9:**
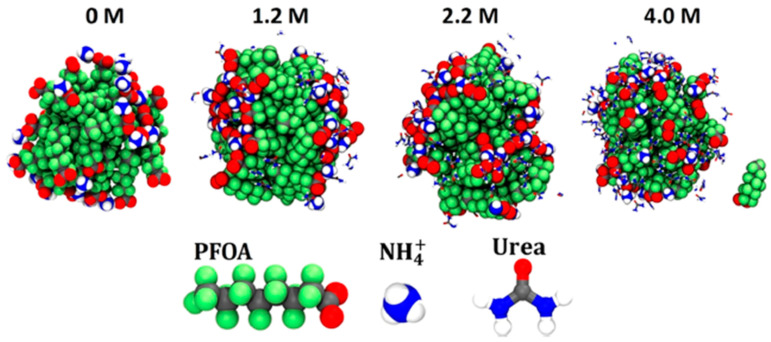
PFOA micelle at different urea concentrations. Reprinted (adapted) with permission from Kancharla et al. [66]. Copyright 2021 American Chemical Society.

Another study, conducted by Kancharla et al. [66], focused on how urea altered the structure of PFOA micelles in an aqueous environment. MD simulations were conducted similarly to the previous study with a number of PFOA and water molecules equaling a concentration of 390 mM, which is again well above CMC concentrations for PFOA. Varying levels of urea were also used throughout the simulations. The singular micelle that formed was shown to compact in the presence of urea. As the concentration of urea increased, the shape of the micelle turned from ellipsoid in water to more spherical at the highest levels of urea. Of note, one PFOA ion dissociated from the rest of the micelle at 4 M urea concentration. Figure 9 shows the PFOA micelle at the various levels of urea concentrations. Across all urea concentrations, urea was located either in the water phase or on the micelle surface itself. This helped to solvate the hydrophobic micelle core by taking the place of some water molecules. Radial distribution function calculations showed that urea strongly binds to the carboxylic head groups of the PFOA with about 1.5 urea molecules in the first coordination shell, which decreased overall electrostatic interactions. Also, as the urea concentration increased, the number of urea molecules in contact with fluorine atoms linearly increased, while the number of water molecules in contact with fluorine decreased. These MD simulations also matched and were consistent with small-angle neutron scattering (SANS) experiments [66].

Kuo et al. [38] examined the morphologies of perfluorosulfonic acid (PFSA) in a water–ethanol solvent mixture using coarse-grain MD for use in catalyst inks for fuel cells. Segments of the structures of the PFSA and the solvent mixture were grouped and assigned as individual entities. The PFSA was assumed to have fully ionized sulfonic acid groups. Varying ethanol concentrations in the solvent were investigated from 0–90 wt% ethanol. In a pure 100% water solvent, the PFSA molecules exhibited a spherical or slightly cylindrical shape. As the ethanol concentration increased up to 50 wt%, the molecules gradually elongated into cylinders. Once the concentration reached 70 wt% ethanol, the cylinders began to uncoil and became highly solvated by ethanol at 90 wt% ethanol. Most of the PFSA particles formed from one chain of PFSA across all concentrations of ethanol with the amphiphilic properties of the charged chain being the driving force for the formation of cylindrical PFSA particles in pure water. The electrostatic interactions between the charged groups were enhanced at small concentrations of ethanol as the dielectric constant of the solvent was decreased. At higher concentrations of ethanol, the surface area increased, causing more ethanol to penetrate the structure, increasing the PFSA/solvent attraction while decreasing the electrostatic interactions between the charged groups [38].

### 2.7. Pyrophyllite Clay

Other soil components, such as hydrated pyrophyllite clay and a humic substance model, were investigated by Luft et al. [67] as potential adsorbent materials for PFAS removal with both taking part in MD simulations. Starting configurations of PFASs began with enough PFAS molecules to correspond to 0.25–0.89 M. Trials were completed after equilibration runs were performed. Umbrella sampling calculations were also conducted, which involved only one PFOS molecule adsorbing onto the surface. Overall, PFAS geometry greatly influenced the degree of adsorption onto the surface. The nonionic monomers of PFASs tended to aggregate quickly into micelles and adsorb onto the surface of the clay within the first 10 ns of the simulation. However, this was not considered true adsorption as the micelles remained spherical and likely would have deformed otherwise.

**Figure 10:**
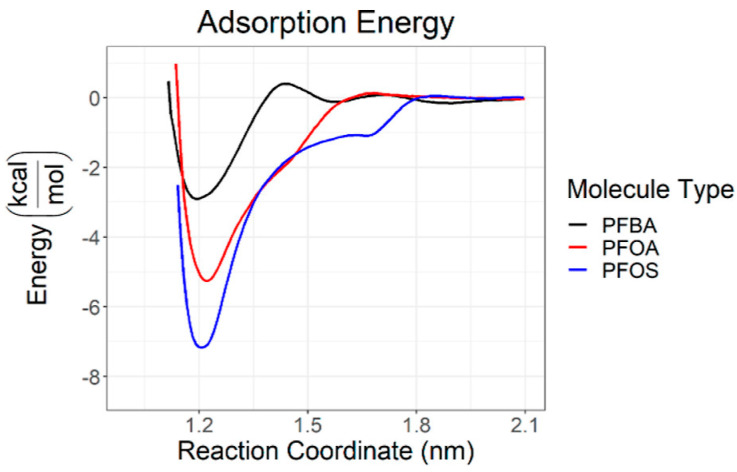
Calculated adsorption energies of perfluorobutanoic acid (PFBA), PFOA, and PFOS as a function of distance from clay surface. Reprinted (adapted) with permission from Luft et al. [67]. Copyright 2022 American Chemical Society.

Of the anionic PFASs examined, perfluorobutanoic acid (PFBA) was the most transient and constantly exchanged with the surface. Anionic PFOA and PFOS existed as aggregates in the simulations and as the binding/desorption processes approached a steady state, they both tended to remain bound to the surface. About 99% of PFOA and about 70% of PFOS remained bound to the surface. As the steady state approached, the anions converted from a micellular shape to an adsorbed, flat monolayer. In this case, the carboxylate head groups of PFOA were able to coordinate with the clay surface more closely as opposed to the sulfonate head groups of PFOS due to steric factors. PFOS, however, did exhibit lower adsorption energy compared with PFOA, as shown in Figure 10, indicating that the coordination is more favorable electronically for PFOS. Favorable PFAS/clay interactions are modulated through hydrophobic interactions with the CF chains. PFOS was shown to be more ordered on the surface than PFOA, as PFOS preferred to lay flat on the surface while PFOA initially interacts with the surface through the CF tails before adopting a flat orientation on the surface. For both PFOA and PFOS, nearly no perceptible barrier to adsorption was determined as the more favorable hydrophobic interactions can occur at much higher distances, resulting in lessened electrostatic effects between the head groups and the slightly anionic clay surface. Nonionic humic substances favorably interact with PFASs at about the same magnitude as pyrophyllite clay [67].

### 2.8. Kaolinite Clay

Loganathan and Wilson [68] reported on the molecular-level insights into the adsorption, structural features, and dynamics of PFASs at the mesopores of kaolinite clay in aqueous environments using MD simulations. Kaolinite surfaces were modeled with siloxane and hydroxyl groups exposed to and interacting with the bulk solution. These surfaces were layered but separated enough so that no interactions between layers occurred. Four different deprotonated PFAS species, modeled at the ppm level, were used and differed in chain length and head group to simulate real-world conditions. The simulations included metal cations that are commonly found in clay mineral deposits and surface water to act as counter ions to the anionic PFAS species. Altogether, these were arranged in a way to enable the preferred adsorption conditions to arise during the simulations. The atomic density profiles indicated that all PFASs adsorb directly onto the hydroxyl-rich areas of the kaolinite surface, while the cations adsorb onto the siloxane areas of the surface, exclusively.

**Figure 11:**
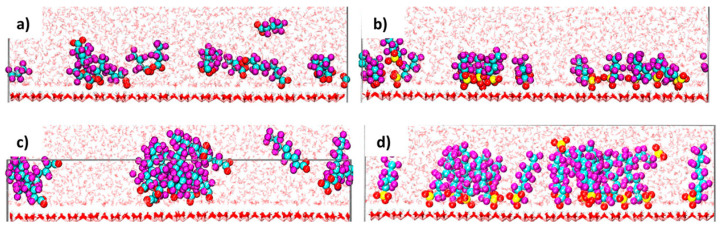
PFAS species interacting with kaolinite surface in water. (**a**) PFBA, (**b**) PFBS, (**c**) PFOA, and (**d**) PFOS with C represented by cyan spheres, F represented by purple spheres, O represented by red spheres, and S represented by yellow spheres. Reprinted (adapted) with permission from Loganathan and Wilson [68]. Copyright 2021 American Chemical Society.

The carboxylate PFASs adsorbed through both oxygen atoms while the sulfonate PFASs adsorbed through only one of the oxygen atoms [59]. Once the oxygen atoms had adsorbed onto the surface, the simulations showed restricted reorientation of the sulfonate groups. Compared with the pyrophyllite clay, the PFASs, when adsorbed onto kaolinite, protrude from the surface and are not parallel. Figure 11 shows how each of the four tested PFASs interact and adsorb onto the kaolinite surface. Only about 25% of the carboxylic PFASs adsorbed onto the surface at a time, while all of the sulfonate PFASs adsorbed, matching the observed experimental isotherms, regardless of whether the PFASs clustered together or not. The short-chain PFASs primarily existed as monomers, as cluster formation was not thermodynamically favorable, while the longer-chain PFASs, like PFOA and PFOS, existed as aggregates. Aggregates typically formed within the first 25 ns of the simulations and, if they did form, they persisted throughout the simulations [68].

### 2.9. Biochar

**Figure 12:**
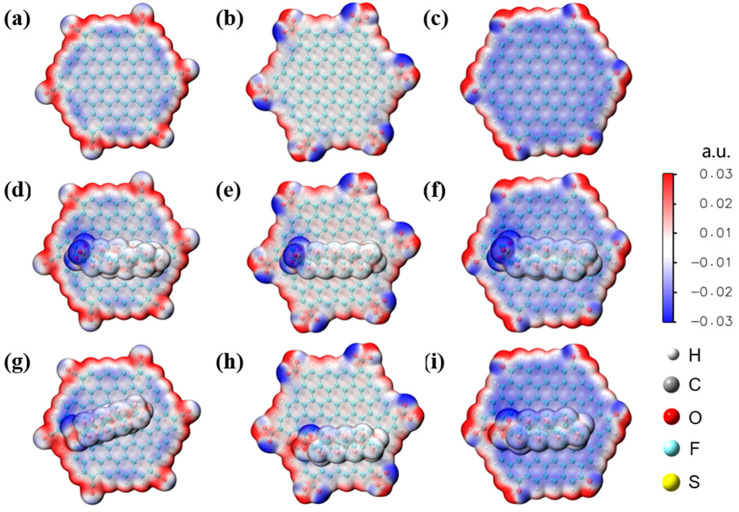
Electrostatic potential maps of (**a**–**c**) graphite-like biochar surfaces along with (**d**–**f**) PFOS adsorption and (**g**–**i**) PFOA adsorption. Functional groups present on the structures include (**a**,**d**,**g**) carbonyl, (**b**,**e**,**h**) carboxylate, and (**c**,**f**,**i**) hydroxyl groups. Reprinted (adapted) with permission from Zhang et al. [69]. Copyright 2023 American Chemical Society.

Biochar is another abundantly available material that has shown effectiveness in removing PFAS contamination. Zhang et al. [69] reported on the electron-scale mechanism of PFAS adsorption by biochar via DFT calculations. The available functional groups on one of the prepared types of biochar, named H-SL, were identified experimentally through Fourier-transform infrared spectroscopy (FTIR) and X-ray photoelectron spectroscopy (XPS), namely carboxylic, hydroxyl, and carbonyl groups. Also, the H-SL was experimentally determined to perform better at lower pH (down to pH 3.0), and it was deduced that this was due to the ionization of the functional groups on the biochar combined with the deprotonation of the PFAS. DFT calculations and the electrostatic potential maps, shown in Figure 12, show that the graphite-like structure of biochar was significantly more negative when the structure was modified with specific functional groups. Taking the macromolecule structure as a whole, the potentials at the positions of the aromatic ring were negative after modification, as shown by the red areas in Figure 12. Graphite modified with carbonyl groups exhibited the highest potential, and the calculated adsorption energies confirmed this. These calculations also showed that PFOA had lower adsorption energies than PFOS, due to the lower electronegativity of the PFOA head group compared with the PFOS head group. For both PFOA and PFOS, the hydrophilic heads were positioned closer to the functional groups on the structure when in the most stable adsorption position, as shown in Figure 12. The long hydrophobic chains were positioned next to the graphite-like rings of the macrostructure. These calculations also shed light on three potential avenues for PFAS adsorption by biochar. Electrostatic interactions between positively charged biochar and deprotonated PFAS head groups, hydrogen bonding between functional groups on the biochar and the PFAS head groups, and hydrophobic interactions between the long hydrophobic CF chains of the PFASs and the biochar all played a part in driving adsorption [69].

### 2.10. Metal–Organic Frameworks

**Figure 13:**
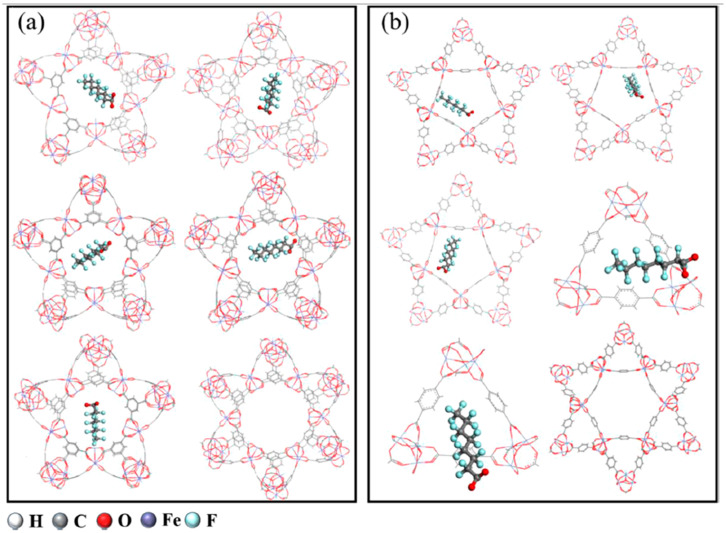
The most stable adsorption configurations of PFOA are metal–organic frameworks (MOFs). (**a**) MIL-100-Fe-PFOA and (**b**) MIL-101-Fe-PFOA. PFOA binds to triangular and pentagonal pores, but not in the hexagonal pores. Reprinted from Yang et al. [70], with permission from Elsevier.

Human-made materials have also shown some promise in being able to effectively remove PFASs from aqueous sources. Yang et al. [70] investigated Fe-based metal–organic frameworks (MOFs), namely Fe-BTC, MIL-100-Fe, and MIL-101-Fe, to determine their potential in adsorbing PFASs. In this work, the authors claimed that DFT calculation involving the entirety of the MOF structure was not possible due to the sheer size of the MOFs. The calculations would be too expensive, computationally. In lieu of using the entire structure, Fe_3_O clusters were used to study the adsorption mechanism of PFOA binding to Fe-based MOFs. The MOFs under investigation have the same Fe_3_O clusters but with different organic ligands surrounding them. Experimentally, the adsorption mechanism indicated that π-CF interactions and Lewis acid/base complexing may be involved. PFOA could also participate in an anion-π interaction with the benzene ring of the MOF or interact with coordinated water or protonated MOF sites. Binding energies and potential structures were calculated that indicate possible mechanisms. Lewis acid/base complexing showed the strongest binding energies as PFOA can displace water coordinated with the Fe_3_O cluster and coordinate with the cluster itself. This result was also confirmed when the electron densities of the systems were plotted. However, under alkaline conditions, this cannot be carried out and the adsorption capacities in these conditions are lessened. Cross and parallel hydrogen bonding can also occur in these complexes, and it was found that parallel hydrogen bonding showed stronger binding energies than cross-hydrogen bonding. To a lesser extent, π-CF and anion-π interactions were also implicated in the adsorption mechanism for PFOA. As shown in Figure 13, PFOA tended to adsorb into the triangular and pentagonal pores of the MOFs and not the hexagonal pores. The more compact space in these areas led to an increased likelihood of favorable interactions [70].

Erkal et al. [71] reported on MOFs functionalized with fluorine atoms to capture PFASs. In this study, the authors used DFT to complete geometry optimizations of the structures. Partial atomic charges were calculated using the REPEAT method, where point charges are fit to reproduce the periodic electrostatic potential of the MOFs. Electrostatic interactions and van der Waals forces were included in the force field used in these calculations. Regarding PFOA, the authors developed a new method where each CF_3_ and CF_2_ group was treated as its own unified unit, and the atoms in the carboxylic group were treated explicitly. These parameters were used in the subsequent MD simulations to validate this method and investigate the MOFs. Monte Carlo simulations were used to calculate Henry’s law coefficients of PFOA and water and to probe the adsorption sites. The calculations of these coefficients are computationally cheap and can provide information about the pore size, geometry, and functionality present in the pores.

In aqueous environments, PFOA is expected to be deprotonated in hydrophilic environments and neutral near hydrophobic sites. Even though these simulations are taking place in aqueous environments, the fluorine functionalization utilized created hydrophobic pockets in the MOFs with no water in the pores; therefore, PFOA was modeled as neutral. Three different fluorine functionalization methods were investigated in total: using fluorinated anions as bridging ligands, substituting ligands in the MOFs with either fluorine or trifluoromethyl, and grafting of perfluorinated alkanes. The fluorine anions as bridging ligands created additional binding sites and water was attracted to these anions. Substitution of ligands to fluorinated groups increased the hydrophobicity but decreased the size of the pores to a point where PFOA could not access the sites effectively. Pore size distribution calculations were completed and it was found that the pore sizes of these MOFs functionalized with trifluoromethyl groups were both less than 6 Å, which is too small for PFOA to access. The grafting method produced MOFs with the highest PFOA/water Henry’s law coefficient ratio resulting in increased affinity for PFOA, but similarly produced pores made it difficult for PFOA to interact efficiently with them. Overall, this study showed that fluorine functionalization can be used to enhance the adsorption affinity of PFASs for MOFs, provided that pore size and accessibility are considerations when developing materials to be used for removing PFASs from aqueous environments [71].

### 2.11. Calixarenes

**Figure 14:**
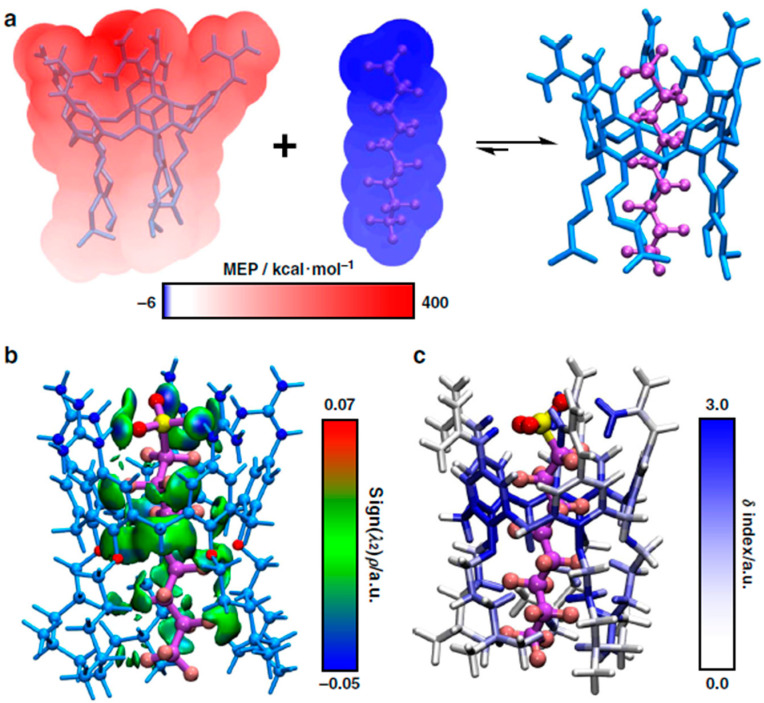
GC5A-6C complexed with PFOS and the (**a**) mapped electrostatic potentials, (**b**) gradient model analysis, and (**c**) complexation contributions. From Zheng et al. [72].

Zheng et al. [72] investigated two different guanidinocalix[5]arenes (GC5A-6C and GC5A-12C) as materials that can simultaneously detect and remove PFASs from water sources. Computationally, only the GC5A-6C arene was investigated due to its complementary size and shape compared with PFOA and PFOS as well as the available bridging interactions. The geometry of GC5A-6C complexed with PFOA and PFOS was optimized using DFT calculations and suggested that the PFASs threaded inside the cavity of GC5A-6C. The molecular electrostatic potential of the GC5A-6C-PFOA complex was also mapped from these calculations onto the van der Waals surfaces of GC5A-6C and PFOS. These results showed that GC5A-6C is electron deficient (red) while PFOS is electron rich (blue), as illustrated in Figure 14a. The gradient model analysis results, as shown in Figure 14b, indicated strong N-H-O hydrogen bonds (blue) between the nitrogen groups and the head group of PFOS. In the same figure, the green areas represent weak C-H-F-C hydrogen bonds between the alkyl chains and the CF chain and van der Waals interactions between the aromatic rings and O with the CF chain. In Figure 14c, the atoms of GC5A-6C were colored to indicate how much they contributed to the adsorption of PFOS into the cavity of GC5A-6C with blue providing the highest contributions and white having nearly no contribution. Here, the nitrogen-containing guanidinium groups and the aromatic rings provided the most contributions toward complexation. The alkyl chains of GC5A-6C also provided some contribution, albeit to a lesser extent. These results, paired with the experimental results and the corresponding arenes, showed that these materials produce a strong binding affinity for PFASs at low concentrations [72].

## 3. Conclusions

Given the power of computers and the accessibility of computational methods, computational approaches in science and engineering are becoming more reliable to help answer emerging questions. Though experimentation remains the gold standard, computational methods can be used to initially investigate a problem and steer the experiments in the right direction or can help confirm and gain a better understanding of how experimental results are interpreted, as noted in several of the studies above. Two common computational methods include DFT and MD and generally provide accurate results if performed correctly. They can be used to analyze systems from different chemical and mechanical perspectives, with DFT focusing on the electron density of the system and the Schrödinger wave equation and MD focusing on the nuclei of the atoms and classical equations of motion. Through these methods, many data points are acquired from systems including binding energies, electrostatic potential maps, optimized spatial configurations, bond distances, etc., and, when combined with experimental data, provide critical insight into the mechanism in question.

Strengths of the methods used in the noted studies include completing the calculations using a sufficient level of detail while maintaining a relatively manageable computational cost. This method of acquiring precise electronic and structural information using DFT and observing system-wide evolution over time from MD requires rigorous attention to the level of theory that is being calculated. Using precise levels of detail in these calculations led to the confirmation of experimental results in several cases, which provides very solid complimentary evidence to highlight the degree of PFAS adsorption and the potential for the material in question. Although providing this evidence is critical to help answer some of the prevailing questions of PFAS remediation, many of the studies still only utilize computation methods as a method of confirming only a few subsets of information, such as the general environment surrounding PFASs, the electronics of an idealized substrate structure, or the interactions solely between PFASs and the substrate under investigation.

Even though it may be more difficult to model correctly and to keep the computational cost low, more focus on PFAS adsorption on various substrates in naturally occurring conditions in contaminated sites may help to judge how well different substrates perform more accurately. Other qualities of effective substrates may also emerge if more real-world conditions are included. Performing simulations with other competing contaminants commonly present at different sites could give more accurate results with respect to material performance in the field. However, this vast increase in complexity may necessitate utilizing coarse-grain MD, or other methods, to simplify the constituents into more manageable segments. Using DFT to obtain very precise structures, energies, and local interactions and using those results to complete MD simulations to acquire information on the entire system may currently be one of the best practices to obtain the necessary amount of evidence to complement experimental results. More robust computational methods, such as AIMD, may provide better information and a more comprehensive picture of how PFAS adsorption occurs.

One emerging question that has helped motivate the development of computational methods is the pervasiveness of growing levels of PFAS contamination in numerous areas of the environment. PFASs have become a large concern for many governing bodies throughout the world as they have been found throughout the food chain and in many water systems around the world, triggering the rapid development of regulations regarding contamination levels. Bioaccumulation across ecosystems, including humans, has raised concerns over the number of potential adverse health effects that have been associated with PFASs. A potential route for remediation includes removing PFASs from the environment using adsorbent materials. Many natural materials such as activated carbon, various types of clay, minerals, and biochar have been shown to be effective at removing PFASs under certain conditions. Some synthetic materials, such as customized MOFs, have also been shown to be effective. In all of these instances, DFT and MD computational methods have provided initial indications of whether or not a material may be favorable toward PFAS adsorption. In addition, calculations have spurred further experimentation using actual materials or have confirmed and supported the experimental evidence by providing information to accurately report on how a material adsorbs PFASs and to what degree. Computational methods, such as DFT and MD, can provide critical information and can help generate more complete answers to emerging questions.

As PFAS contamination continues to increase worldwide, ideal substrates to be used for PFAS remediation may have some of the characteristics that the DFT and MD results above have highlighted. Materials such as clay and AC, which are very common and cost-effective, provide a good starting point for building the ideal substrate to address the widespread PFAS contamination issue. Some materials such as MOFs and calixarenes may be too expensive to scale up to the amounts needed. Materials that exhibit fully positively charged head groups separated by large enough hydrophobic areas tend to have the most favorable interaction with various PFASs. Chitosan, biochar, and clay meet these criteria, especially at lower pH levels, as the positively charged head groups of the substrates interact with the negatively charged head groups of the PFASs through electrostatic interactions and the hydrophobic surfaces of the substrates interact with the hydrophobic CF chains of the PFASs. The size of the components and distances between the charged groups also play a role in how well the adsorption proceeds; as shown above, if there is not enough room for the PFAS to fit in nicely to fully interact with the substrate surface, this hampers the adsorption and makes the material less efficient. To maximize the availability of substrate material to effectively carry out adsorption, materials with high surface areas would be ideal. MOFs, even though would be relatively expensive to deploy on a large scale, exhibit a high surface area that would allow for a large amount of PFASs to adsorb into the structure. Given that PFASs tend to form micelles in aqueous media, tailoring the size of the PFAS micelles may help drive the adsorption of larger amounts of PFASs more quickly into pores of material. Also, being able to control the size may help to reduce pore blocking and extend the life of adsorbent materials. Chitosan—or similar materials—may be best suited for next-generation, large-scale PFAS remediation as this material meets nearly all of the best-case criteria explained through DFT and MD calculations described above.

## 4. Future Directions

Computational research on the adsorption and interaction of PFASs with a variety of substrates has the potential to provide valuable insights into the design of strategies for PFAS remediation, including the development of new computational tools and approaches that can help researchers better understand the behavior of PFASs in the environment and in the development of new technologies for the remediation of PFAS-contaminated sites. For example, the use of machine learning algorithms to analyze large datasets can help identify patterns and relationships that may not be immediately apparent from the data. While much research has been conducted on the adsorption and interaction of PFASs with common substrates, there is still much to be learned about the behavior of PFASs with other substrates. Finally, collaboration between researchers from different fields can help drive innovation and progress in the field of PFAS research. By working together, researchers can combine their expertise and develop new approaches to address this critical problem. These are just a few possible directions for computational research on the adsorption and interaction of PFASs with a variety of substrates using DFT and MD that can help lead to solutions to this critical problem that impacts the environment and human health.

## Data Availability

No new data were created or analyzed in this study. Data sharing is not applicable to this article.
